# Maternal and Infant Health Outcomes in US-Born and Non–US-Born Black Pregnant People in the US

**DOI:** 10.1001/jamanetworkopen.2024.51693

**Published:** 2024-12-26

**Authors:** Mariah Jiles, Ndola Prata, Kim G. Harley

**Affiliations:** 1 Center of Excellence in Maternal, Child and Adolescent Health, University of California, Berkeley; 2 Bixby Center for Population Health and Sustainability, School of Public Health, University of California, Berkeley; 3 Wallace Center for Maternal, Child, and Adolescent Health, School of Public Health, University of California, Berkeley

## Abstract

**Importance:**

With disparate Black maternal health outcomes in the US and a steadily expanding non–US-born Black population, it is beneficial to investigate Black maternal health outcomes by country of origin.

**Objective:**

To compare the prevalence of maternal morbidity and infant birth outcomes between US-born and non–US-born Black populations in the US.

**Design, Setting, and Participants:**

This cross-sectional study included all registered hospital births in the US from the 2021 National Vital Statistics Systems (NVSS) Natality Data. Eligible patients identified as Black, excluding those who were younger than 15 years, gave birth to twins or multiple infants, gave birth outside of a hospital, or were missing data for maternal morbidity, nativity, or study covariates.

**Exposures:**

Maternal birthplace, characterized as born within the US or born outside of the US.

**Main Outcomes and Measures:**

Five maternal morbidities (ie, maternal transfusion, perineal laceration, ruptured uterus, unplanned hysterectomy, admission to intensive care unit) and two birth outcomes (low birthweight, preterm birth). Logistic regression analyses were utilized to calculate the associations of maternal morbidity and adverse birth outcome with maternal birthplace.

**Results:**

Of a total 3 669 928 registered births in the US in 2021, 499 409 births to mothers who identified as Black were included in analysis; the majority of individuals were aged 20 to 24 years (117 173 [23.5%]), 25 to 29 years (142 890 [28.6%]), or 30 to 34 years (123 485 [24.7%]). Maternal birthplace in the US was associated with a decreased odds of experiencing any maternal morbidity (4411 of 403 822 births [1.1%]) compared with Black people born outside of the US (1593 of 95 587 births [1.7%]) (aOR, 0.67; 95% CI, 0.62-0.71). US-born Black people were significantly less likely to experience 3 of 5 maternal morbidities compared with non–US-born Black people: maternal transfusion (aOR, 0.87; 95% CI, 0.78-0.97), perineal laceration (aOR, 0.43; 95% CI, 0.39-0.48), and ruptured uterus (aOR, 0.63; 95% CI, 0.45-0.89). US-born Black people also had a nonsignificant decrease in odds for unplanned hysterectomy (aOR, 0.77; 95% CI, 0.56-1.05) and admission to intensive care unit (aOR, 0.92; 95% CI, 0.79-1.07). However, they were at increased odds of experiencing key adverse infant health outcomes, including low birthweight (aOR, 1.62; 95% CI, 1.58-1.67) and preterm birth (aOR, 1.51; 95% CI, 1.47-1.55).

**Conclusions and Relevance:**

In this cross-sectional study, significant differences were observed in odds of maternal morbidity and birth outcomes by maternal birthplace within the Black pregnant population in the US. This suggests the need for increased assessment of nativity in Black maternal health research and specific strategies to reduce morbidity for non–US-born populations.

## Key Points

**Question:**

What is the association of maternal morbidity occurrence with maternal nativity for Black people in the US?

**Findings:**

In this cross-sectional study of 499 409 births to mothers who identified as Black, non–US-born individuals experienced elevated odds of maternal morbidity compared with those born within the US, while still experiencing lower odds of giving birth to a baby at low birthweight or giving birth preterm.

**Meaning:**

Future public health data collection and research efforts should consider disaggregation by birth country or ethnicity to accommodate the diversity of the Black American population and prevent the obfuscation of potential health inequities.

## Teaser

This cross-sectional study compares the risk of maternal morbidity and adverse birth outcomes among Black US individuals by maternal birthplace, categorized as within the US vs outside of the US.

## Introduction

Currently, Black pregnant people in the US are 3 times more likely to die from pregnancy-related causes than their White counterparts.^1^ While these numbers are alarming, maternal deaths remain a relatively rare phenomenon. Each year, an estimated 700 people will die during pregnancy or in the following year, compared with some 50 000 people who will experience a severe maternal morbidity—an unexpected outcome of labor that can result in significant health consequences, both short-term and long-term.^2,3,4^ Due to the salience of the Black maternal health crisis in the US and the prevalence of maternal morbidities, it is imperative to further examine the experiences of Black pregnant individuals with maternal morbidities.^1,5^

While there has been considerable research on the health of Black pregnant people in the US, little research has articulated how the diversity of Black American experiences is reflected in maternal health outcomes. In research, Black is treated as a homogenous identity, despite the variety of different experiences inherent in this group depending on ethnicity. African American (individuals who descend from enslaved Africans in the US), Afro-Caribbean, Afro-Latine, and African populations in the US, while all being aggregated into one racial group, have been found to have many differing health behaviors and outcomes.^6,7,8^ Research has shown inequities in the outcomes of US-born and non–US-born Black people, with US-born Black populations tending to have worse prepregnancy nutritional status, worse prenatal health behaviors, and an increased likelihood of giving birth to babies with lower birth weights compared with non–US-born Black populations in the US.^6,7,8^

**Figure 1.**
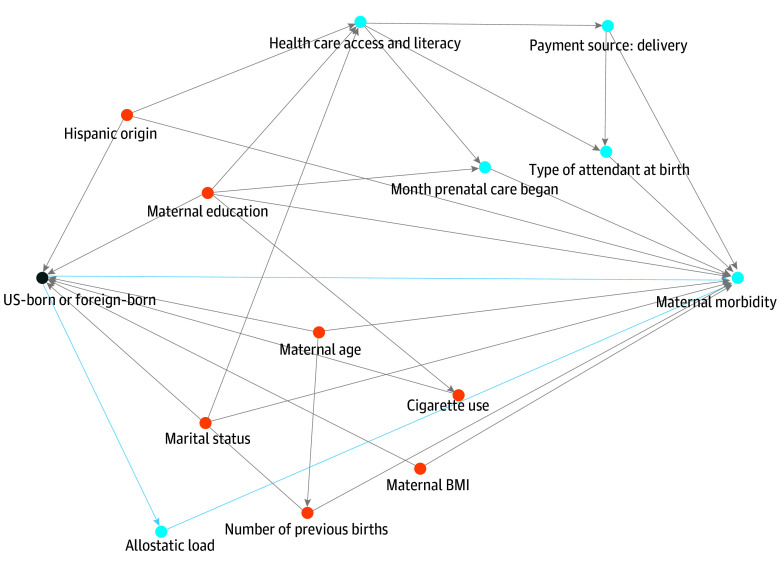
Directed Acyclic Graph (DAG) of Association Between Mother’s Birth Country, Maternal Morbidity, and Covariates^10^ US-born or non–US-born indicates the exposure, and maternal morbidity the outcome; orange dots, ancestor of the exposure and outcome (covariate); blue dots, ancestor of the outcome. BMI indicates body mass index (calculated as weight in kilograms divided by height in meters squared).

One explanation for these observed poorer outcomes among US-born Black women is the weathering hypothesis—the theory that chronic exposure to racism has cumulative effects that lead to accelerated aging of the body—which posits that US-born Black populations who have been in the US for significantly longer periods of time could be more susceptible to these harmful experiences and health outcomes.^9^ However, other factors, including interactions with systems of health and power in the US (ie, social determinants of health) can also contribute to differential health outcomes between US and non–US-born Black individuals (Figure 1).^11^ Considerations such as experience navigating the US health care system (often referred to as “health literacy”), language barriers, and health insurance coverage, among other societal factors, can impact the ability of those of either nativity status to achieve their highest level of health.

Despite research showing associations of US nativity with poorer birth outcomes, few studies have examined how risk of maternal morbidity differs between US-born and non–US-born Black people. Exploration into the association of ethnicity on Black maternal birth outcomes is timely due to the steady expansion of the non–US-born Black populations in the US. The number of non–US-born Black people living in the US more than tripled from 1980 to 2019, growing from 800 000 in 1980 to 4.6 million in 2019, and this population is projected to grow to a sizable 9.5 million by 2060.^12^ Along with the 12% of Black individuals who themselves were born outside of the US, another 9% are second-generation US residents with at least 1 non–US-born parent. As the non–US-born Black population continues to grow in the US, it will be increasingly advantageous to distinguish between different Black communities in research.

The purpose of this study is to compare maternal morbidity and key infant health outcomes between the US-born and non–US-born Black populations in 2021. We hypothesized that US-born Black populations experience more maternal morbidities than non–US-born Black populations in the US based on this group’s observed higher rates of low birth weight and preterm birth and on extended exposure to experiences of racism that contribute to intergenerational weathering. We seek to add to current research on the Black maternal health crisis that accommodates the diversity of Black American identities in the US and its subsequent association with health outcomes, as well as to further examine the prevalence of maternal morbidities in birthing populations.

## Methods

Public-use National Vital Statistics System (NVSS) birth data from 2021 were used for this study. NVSS provides the most complete data on births in the US by utilizing birth certificates of all births in the country each year.^13,14^ In the US, all states require birth certificates to be filed for all births and federal law mandates the collection and publication of birth data.^13^ The NVSS is the result of federal compilation of birth data in the US.^13^ More than 99% of all births occurring in the US are registered.^15^ A total of 3 669 928 births were reported to the NVSS in 2021.

**Figure 2.**
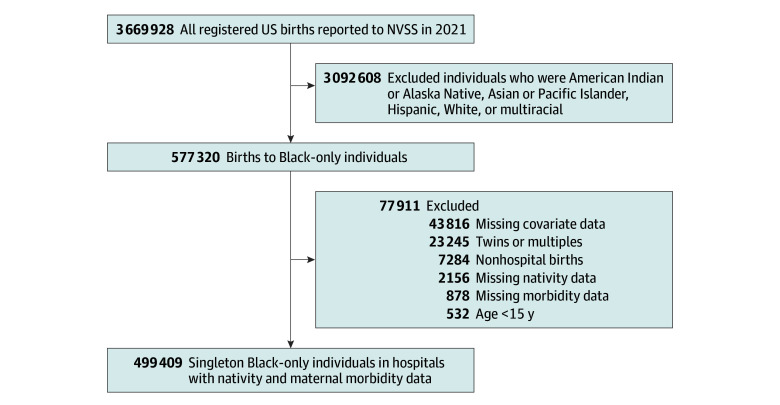
Analytical Sample Selection Process NVSS indicates National Vital Statistics Systems.

First, individuals who identified as a race other than Black or who reported more than 1 race were excluded (Figure 2). Next, we excluded individuals who gave birth to twins or multiple infants, gave birth outside of a hospital, or were younger than 15 years old. Finally, individuals who were missing maternal nativity, overall maternal morbidity composite measure, or covariate data were removed. The most commonly missing covariates were marital status, body mass index (BMI; calculated as weight in kilograms divided by height in meters squared) and education status. Due to our utilization of public-use, deidentified data, this research was considered exempt from review and informed consent requirements by the University of California, Berkeley Committee for the Protection of Human Subjects. This report follows the Strengthening the Reporting of Observational Studies in Epidemiology (STROBE) reporting guidelines for cross-sectional observational studies.

Data for mother’s country of birth were collected using the 2003 revision of the US Standard Certificate of Live Birth. On the form, the mother’s birthplace was requested, asking for either the state, territory, or country. In our analysis, maternal birthplace was coded as a dichotomous variable—mothers were either born within the US vs born outside of the US or in a US territory.

Five different maternal morbidities were separately identified in the NVSS data using a checkbox format: maternal transfusion, third- or fourth-degree perineal laceration, ruptured uterus, unplanned hysterectomy, and admission to intensive care unit (ICU). We analyzed each outcome separately as dichotomous variables as well as examining a dichotomous variable for the occurrence of any of the 5 maternal morbidities. To confirm previous studies, we also examined associations of maternal nativity with birth outcomes. Low birthweight was classified as under 2500 g and preterm birth as birth before 37 weeks of pregnancy.^16^ All infant variables were treated as dichotomous.

Covariates for this study were selected a priori using a directed acyclic graph (DAG)^17^ created by our study team to clarify and visualize our understanding and assumptions of causal relationships between variables (Figure 1). Our covariates included maternal age, maternal education, Hispanic origin, marital status, maternal BMI, month prenatal care began, payment source for delivery (Medicaid, private insurance, self-pay, other), type of attendant at birth, cigarette smoking during pregnancy, and number of previous births.

### Statistical Analysis

We first performed bivariate analyses utilizing χ^2^ tests to compare characteristics of US- and non–US-born mothers. Next, we conducted multivariable logistic regression to examine associations between maternal country of birth and maternal morbidity and birth outcomes, controlling for covariates. In sensitivity analyses, we re-did our analyses twice: (1) expanding the population to include multiracial individuals who identified as Black in addition to 1 or more other races and (2) limiting our dataset to nulliparous women only. Results were designated as significant using a 2-tailed α = .05. All statistical analyses were performed using the statistical software Stata/BE version 17.0 (StataCorp).

## Results

A total of 499 409 singleton births to Black individuals were included for analysis, 403 822 births from US-born and 95 587 from non–US-born Black people. The majority of individuals were aged 20 to 24 years (117 173 [23.5%]), 25 to 29 years (142 890 [28.6%]), or 30 to 34 years (123 485 [24.7%]) (Table 1). Approximately two-thirds of the sample (344 040 individuals [68.9%]) had graduated high school or earned a General Educational Development test certification while 94 680 (19.0%) had earned an associate’s degree, college degree, or higher. Most of the population (350 679 individuals [70.2%]) was unmarried at the time of birth and more than half (320 873 [64.3%]) utilized Medicaid as the payment source for their delivery. Overall, 410 917 births (82.3%) were attended by a medical doctor (MD) and 48 123 (9.6%) had a certified nurse midwife or certified midwife present at birth. Maternal morbidity was observed in 6004 births (1.2%), with maternal transfusion (2695 [0.5%]) and third- or fourth-degree perineal laceration (2127 [0.4%]) being the most reported morbidities.

**Table 1.** Selected Characteristics of Singleton Black Births in the US by Maternal Nativity

Characteristic	Births, No. (%)			*P* value^a^
	Total (N = 499 409)	US-born birthing person (n = 403 822)	Non–US-born birthing person (n = 95 587)	
Maternal age, y				
15-19	31 896 (6.4)	29 947 (7.4)	1949 (2.0)	<.001
20-24	117 173 (23.5)	106 028 (26.3)	11 145 (11.7)	
25-29	142 890 (28.6)	119 968 (29.7)	22 922 (24.0)	
30-34	123 485 (24.7)	93 315 (23.1)	30 170 (31.6)	
35-39	65 757 (13.2)	43 663 (10.8)	22 094 (23.1)	
40-44	16 948 (3.4)	10 314 (2.6)	6634 (6.9)	
≥45	1260 (0.3)	587 (0.2)	673 (0.7)	
Maternal education				
Did not graduate high school	60 689 (12.1)	43 500 (10.8)	17 189 (18.0)	<.001
High school graduate or some college	344 040 (68.9)	292 235 (72.4)	51 805 (54.2)	
College graduate	94 680 (19.0)	68 087 (16.9)	26 593 (27.8)	
Hispanic origin				
No	451 597 (90.4)	377 747 (93.5)	73 850 (77.3)	<.001
Yes	47 812 (9.6)	26 075 (6.5)	21 737 (22.7)	
Marital status				
Single	350 679 (70.2)	311 766 (77.2)	38 913 (40.7)	<.001
Married	148 730 (29.8)	92 056 (22.8)	56 674 (59.3)	
Maternal BMI				
Underweight (<18.5)	13 499 (2.7)	11 445 (2.8)	2054 (2.2)	<.001
Normal weight (18.5-24.9)	147 968 (29.6)	117 545 (29.1)	30 423 (31.8)	
Overweight (25.0-29.9)	135 671 (27.2)	102 386 (25.4)	33 285 (34.8)	
Obese (30 to 40)	202 271 (40.5)	172 446 (42.7)	29 825 (31.2)	
Prenatal care start, gestational age, mo				
1-3	341 322 (68.4)	281 733 (69.8)	59 589 (62.3)	<.001
4-6	104 621 (21.0)	81 005 (20.1)	23 616 (24.7)	
7-final	28 037 (5.6)	20 091 (5.0)	7946 (8.3)	
No prenatal care	15 110 (3.0)	12 731 (3.2)	2379 (2.5)	
Unknown or not stated	10 319 (2.1)	8262 (2.1)	2057 (2.2)	
Payment for delivery				
Medicaid	320 873 (64.3)	267 419 (66.2)	53 454 (55.9)	<.001
Private insurance	149 565 (30.0)	118 289 (29.3)	31 276 (32.7)	
Self-pay	10 555 (2.1)	3853 (1.0)	6702 (7.0)	
Other	15 393 (3.1)	12 013 (3.0)	3380 (3.5)	
Unknown	3023 (0.6)	2248 (0.6)	775 (1.0)	
Attendant at birth				
MD	410 917 (82.3)	333 591 (82.6)	77 326 (80.9)	<.001
DO	34 718 (7.0)	28 020 (6.9)	6698 (7.0)	
CNM or CM	48 123 (9.6)	37 406 (9.3)	10 717 (11.2)	
Other	5651 (1.1)	4805 (1.2)	846 (0.9)	
Cigarette use				
No	481 026 (96.3)	385 755 (95.5)	95 271 (99.7)	<.001
Yes	18 383 (3.7)	18 067 (4.5)	316 (0.3)	
Parity				
1	183 563 (36.8)	153 548 (38.0)	30 015 (31.4)	<.001
2	141 747 (28.4)	112 738 (27.9)	29 009 (30.4)	
3	89 635 (18.0)	70 033 (17.3)	19 602 (20.5)	
4	45 490 (9.1)	36 277 (9.0)	9213 (9.6)	
5	20 627 (4.1)	16 678 (4.1)	3949 (4.1)	
6	9431 (1.9)	7636 (1.9)	1795 (2.0)	
7	4442 (1.0)	3566 (1.0)	876 (1.0)	
≥8	4474 (1.0)	3346 (1.0)	1128 (1.2)	
Any maternal morbidity				
Yes	6004 (1.2)	4411 (1.1)	1593 (1.7)	<.001
No	493 405 (98.8)	399 411 (98.9)	93 994 (98.3)	
Maternal transfusion				
Yes	2695 (0.5)	2126 (0.5)	569 (0.6)	.009
No	496 714 (99.5)	401 696 (99.5)	95 018 (99.4)	
Perineal laceration				
Yes	2127 (0.4)	1370 (0.3)	757 (0.8)	<.001
No	497 282 (99.6)	402 452 (99.7)	94 830 (99.2)	
Ruptured uterus				
Yes	214 (<0.1)	150 (<0.1)	64 (0.1)	<.001
No	499 195 (>99.9)	403 672 (>99.9)	95 523 (99.9)	
Unplanned hysterectomy				
Yes	245 (<0.1)	172 (<0.1)	73 (0.1)	<.001
No	499 164 (>99.9)	403 650 (>99.9)	95 514 (99.9)	
Admittance to ICU				
Yes	1223 (0.2)	948 (0.2)	275 (0.3)	.003
No	498 186 (99.8)	402 874 (99.8)	95 312 (99.7)	

Abbreviations: BMI, body mass index (calculated as weight in kilograms divided by height in meters squared); CM, certified midwife; CNM, certified nurse–midwife; ICU, intensive care unit; MD, medical doctor.

^a^ *P* values from χ^2^ test.

Non–US-born individuals differed significantly from US-born on all characteristics (Table 1). Non–US-born individuals were older (eg, ages 30 to 34 years: 30 170 of 95 587 births [31.6%] vs 93 315 of 403 822 births [23.1%]) and represented a higher percentage of college graduates (26 593 of 95 587 births [27.8%] vs 68 087 of 403 822 births [16.9%]) than US-born Black individuals. Groups differed considerably on marital status, with 59.3% (56 674 of 95 587 births) of the non–US-born population being married at time of birth compared with 22.8% (92 056 of 403 822 births) of the US-born population. In addition, US-born individuals tended to start prenatal care earlier, with 69.8% (281 733 of 403 822 births) beginning prenatal care in the first to third month of pregnancy compared with 62.3% (59 589 of 95 587 births) of non–US-born people. US-born individuals were more likely to use Medicaid as the payment source for delivery (267 419 of 403 822 births [66.2%]) compared with non–US-born individuals (53 454 of 95 587 births [55.9%]). Notably, 7.0% of non–US-born people (6702 of 95 587 births) reported self-paying for delivery, which is often an indication of being uninsured, compared with 1.0% of US-born people (3853 of 403 822 births).

Maternal birthplace in the US was associated with decreased odds in occurrence of any maternal morbidity assessed in this study after controlling for covariates (adjusted odds ratio [aOR], 0.67; 95% CI, 0.62-0.71) (Table 2). When examining specific maternal morbidity diagnoses, maternal birthplace in the US was associated with significantly decreased odds of each discrete morbidity: experiencing maternal transfusion (aOR, 0.87; 95% CI, 0.78-0.97), perineal laceration (aOR, 0.43; 95% CI, 0.39-0.48), and ruptured uterus (aOR, 0.63; 95% CI, 0.45-0.89). US birthplace also had nonsignificant decreases in odds of unplanned hysterectomy (aOR, 0.77; 95% CI, 0.56-1.05) and admission to an ICU (aOR, 0.92; 95% CI, 0.79-1.07).

**Table 2.** Adjusted Odds Ratios (aOR) for Occurrence of Maternal Morbidities by Maternal Birth Country Among Black Birthing People in the US

Maternal morbidity^b^	aOR (95% CI)^a^	
	US-born birthing person	Non–US-born birthing person
Any maternal morbidity	0.67 (0.62-0.71)	1 [Reference]
Maternal transfusion	0.87 (0.78-0.97)	1 [Reference]
Perineal laceration	0.43 (0.39-0.48)	1 [Reference]
Ruptured uterus	0.63 (0.45-0.89)	1 [Reference]
Unplanned hysterectomy	0.77 (0.56-1.05)	1 [Reference]
Admission to ICU	0.92 (0.79-1.07)	1 [Reference]

^a^ Adjusted for: maternal age, maternal education, Hispanic origin, marital status, maternal body mass index, month prenatal care began, payment source for delivery, attendant at birth, cigarette use during pregnancy and parity.

^b^ Due to missing data, totals of births included for analysis by outcome were 499 409 for maternal transfusions, 494 935 for perineal laceration, 495 131 for ruptured uterus, 499 409 for unplanned hysterectomy, and 499 409 for intensive care unit (ICU) admittance.

Conversely, US-born Black people were more likely to experience adverse infant birth outcomes. US-born pregnant people had increased odds of having a baby with low birthweight (aOR, 1.62; 95% CI, 1.58-1.67) and preterm birth (aOR, 1.50; 95% CI, 1.47-1.55).

In sensitivity analyses inclusive of multiracial Black individuals, results were largely the same, with US-born women displaying significantly decreased odds of overall maternal morbidity as well as decreased odds of experiencing each of the five morbidities independently, but increased odds of low birth weight and preterm birth (eTable 1 in Supplement 1). These associations were also robust when we examined only nulliparous people, however results for ruptured uterus and unplanned hysterectomy were no longer statistically significant (eTable 2 in Supplement 1).

## Discussion

Contrary to our hypothesis, we found that US-born Black pregnant people are at decreased odds of maternal morbidity compared with non–US-born Black individuals. Conversely, and consistent with other studies, we found that US-born Black pregnant people are at increased odds of delivering a low birthweight or preterm infant. This study is the first to our knowledge that explores maternal morbidity outcomes between US-born Black people and Black people born outside of the US.

Although the results were unexpected, preexisting conditions such as hypertension, diabetes, and others could lead to non–US-born Black women being at higher odds of experiencing maternal morbidities. It is also critical to consider structural and societal factors that affect immigrant health and could contribute to the differential odds of experiencing maternal morbidity. Adjusting to living in a new country has been found to alter social and behavioral health factors for any immigrant group.^18^ Outside of health insurance coverage, which was controlled for in our analysis, gaps in health care access and quality could persist. Compared with the overall US immigrant population, Black immigrants to the US have been found more likely to be US citizens and to speak English at a proficient level, with 74% of Black immigrants over 5 years old speaking English proficiently.^19^ For individuals who speak a language other than English, there is a potential for linguistic barriers to affect the quality and type of health care they receive. For Black immigrants from the African continent who speak a language other than English, gaps in care remain.^20^ As the African continent comprises 54 countries, there could be a high degree of language variability within one country alone.^20^

Discrimination within the health care system could also be a potential factor affecting the experiences of non–US-born Black people. African immigrants have reported being treated differently in health care settings than other patients based on their accents and cultural mode of dress.^20^ Again, while this is not representative of the experience of all Black immigrants living in the US, it could provide useful insight into the potential impact of structural factors on maternal health outcomes.

Results are consistent with research finding US-born Black individuals at a higher risk for preterm birth and low birthweight. In a study examining Florida birth certificates from 1971 to 2015, non–US-born Black women had lower prevalence of having a baby with low birthweight (7.8% compared with 11.8% for US-born Black women). Within one generation, this disparity disappeared, with low birthweight prevalence in the daughters of non–US-born women rising to 12.2% compared with 13.1% for US-born Black women.^21^ This may suggest that multiple social and structural mechanisms in the US are at play, influencing the health of Black birthing people.

A study conducted by the Pew Research Center found that nearly half of all US-born Black people interviewed reported they have few things or nothing in common with Black immigrants living in the US, compared with 14% who state they have everything or most things in common with them.^22^ While these data are based on self-perceived experiences, it could be reflective of a larger reality of differences in the social experiences of Black individuals living in the US. Considering the weathering hypothesis that details how chronic exposure to stressors is associated with worsened physical health, differences in the social experiences between Black populations could be associated with inequities in maternal health outcomes.

Regarding differences between the 2 populations in risk of maternal vs infant health outcomes, it is possible that a separate mechanism could be at play. If one looks at pregnancy as a chronic or ongoing experience occurring over numerous months, infant health could be more susceptible to the weathering body or experiences of racism or discrimination experienced by the birthing person. This could contribute to the finding that US-born Black people have higher rates of adverse infant health outcomes compared with non–US-born populations. Maternal morbidity could be seen as a more acute or sudden occurrence. A birthing person could have a healthy pregnancy up until time of delivery and an occurrence at the hospital-level could be associated with a specific morbidity.

Results from this study provide evidence of the differences in maternal morbidity, preterm and low birthweight occurrence among Black birthing people in the US, some of which has not been previously shown. For clinicians, the results of this study could bring greater awareness to differential maternal health outcomes within the Black community based on nativity status. In clinical care, this could manifest as refining language justice initiatives to meet the needs of pregnant people in the community, who may be non-English and non-Spanish speakers, as well as additional trainings in culturally competent care that accommodates the experience of birthing people who are Black but not African American. For policymakers, the results of this study could lead to increased engagement with non–US-born Black (immigrant) communities to examine potential barriers to maternal health care that is being experienced but may not currently be addressed in a meaningful way. And finally, for researchers, this study can draw closer attention to the need for greater specificity (disaggregation) when collecting data and reporting maternal and infant health outcomes of Black individuals in a way that works alongside community members, seeks insight from those who are having their data collected, and is attuned to the current realities of being an immigrant to the US.^23^

### Limitations

There are limitations in the interpretation of our study results. We were limited to the 5 morbidities monitored by NVSS, which exclude additional, less severe morbidities that could have been experienced by our population (eg, puerperal infection, hemorrhage). We were unable to examine the amount of time that non–US-born people spent in the US or the association of acculturation with maternal morbidity. From this dataset we also were unable to ascertain the geographic location of each individual or where they gave birth and resulting associations with maternal morbidity occurrence. In a Pew Research Study utilizing 2019 American Community Survey data, 42% of Black immigrants in the US were found to live in the South, compared with 36% living in the Northeast, 11% in the Midwest, and 10% in the West.^22^ Further exploration is needed to examine whether regional differences in birth location within the US is associated the occurrence of maternal morbidities for Black birthing people. We were also unable to examine potential language barriers in participants, and whether English was their first language. A lack of English proficiency has been associated with inequities in a variety of health outcomes in previous studies, including access to preventative care and health status.^24^ It would have been beneficial to examine how language barriers are linked to adverse maternal health outcomes for non–US-born populations in future studies. In utilizing publicly available data, we were unable to access what country each parent was born in and instead had to rely on a broad non–US-born maternal designation. Individuals immigrate to the US under differing circumstances. An individual immigrating due to violence, war, or other forms of persecution may have an entirely different set of economic and social circumstances than individuals coming to the US for educational or professional opportunities. Therefore, it would be beneficial to see differences in maternal morbidity rates by region of birth. Finally, some studies have found inaccuracies in birth certificate data, noting that there could be variations in subgroup data quality, which could influence the findings of this study.^25^

## Conclusions

Black maternal health in the US is an area of critical importance with higher mortality than any other race or ethnicity. Analysis of maternal morbidity reveals that non–US-born Black people have significantly higher odds of experiencing severe morbidities than their US-born counterparts. In contrast, US-born Black individuals have disproportionately elevated odds of adverse birth outcomes such as preterm birth and low birthweight. This suggests the need for increased assessment of nativity in Black maternal health research and specific strategies to reduce morbidity for non–US-born populations.

## Notes

Supplement 1. **eTable 1.** Adjusted Odds Ratio for Occurrence of Maternal Morbidities by Maternal Birth Country Among All Black (Multiracial Included) Birthing People in the US, National Vital Statistics System Birth Data, 2021 **eTable 2.** Adjusted Odds Ratios for Occurrence of Maternal Morbidities by Maternal Birth Country Among Nulliparous Black-only Birthing People in the US, National Vital Statistics System Birth Data, 2021

Supplement 2.
Data Sharing Statement
