# Supplementary material for: Maternal and Infant Health Outcomes in US-Born and Non–US-Born Black Pregnant People in the US
Source: JAMA Netw Open. 2024 Dec 26;7(12):e2451693. doi: 10.1001/jamanetworkopen.2024.51693 (PMC11672153; doi:10.1001/jamanetworkopen.2024.51693)
Supplement: Supplement 1. — eTable 1. Adjusted Odds Ratio for Occurrence of Maternal Morbidities by Maternal Birth Country Among All Black (Multiracial Included) Birthing People in the US, National Vital Statistics System Birth Data, 2021 eTable 2. Adjusted Odds Ratios for Occurrence of Maternal Morbidities by Maternal Birth Country Among Nulliparous Black-only Birthing People in the US, National Vital Statistics System Birth Data, 2021 [file jamanetwopen-e2451693-s001.pdf]

Supplemental Online Content

Jiles M, Prata N, Harley KG. Maternal and infant health outcomes in US-born and foreign-born Black pregnant people in the US. *JAMA Netw Open.* 2024;7(12):e2451693.
doi:10.1001/jamanetworkopen.2024.51693

eTable 1. Adjusted Odds Ratio for Occurrence of Maternal Morbidities by Maternal Birth Country Among All Black (Multiracial Included) Birthing People in the US, National Vital Statistics System Birth Data, 2021

eTable 2. Adjusted Odds Ratios for Occurrence of Maternal Morbidities by Maternal Birth Country Among Nulliparous Black-only Birthing People in the US, National Vital Statistics System Birth Data, 2021

This supplemental material has been provided by the authors to give readers additional information about their work.

© 2024 Jiles M et al. *JAMA Network Open.*

eTable 1: Adjusted[a] odds ratio for occurrence of maternal morbidities by maternal birth country among all Black (multiracial included) birthing people in the U.S., National Vital Statistics System Birth Data, 2021. (n=544,606)

Maternal Birthplace	Maternal Morbidity	Maternal Transfusion	Perineal Laceration	Ruptured Uterus	Unplanned Hysterectomy	Admittance to ICU	Low birthweight	Preterm birth
	aOR(95% CI)	aOR(95% CI)	aOR(95% CI)	aOR(95% CI)	aOR(95% CI)	aOR(95% CI)	aOR (95% CI)	aOR (95% CI)
U.S.-born	0.66 (0.62-0.70)	0.85 (0.77-0.94)	0.45 (0.41-0.50)	0.65 (0.46-0.91)	0.80 (0.58-1.1)	0.89 (0.77-1.0)	1.57 (1.53-1.61)	1.48 (1.44-1.52)
Foreign-Born	Reference	Reference	Reference	Reference	Reference	Reference	Reference	Reference

[a] Adjusted for: maternal age, maternal education, Hispanic origin, marital status, maternal BMI, month prenatal care began, payment source for delivery, attendant at birth, cigarette use during pregnancy and parity

[b] Due to missing data, n= 544,490 for maternal morbidity, maternal transfusion, unplanned hysterectomy and ICU admittance; n= 539,822 for perineal laceration; n=539,783 for ruptured uterus; . n=544,145 for low birthweight and n=544,352 for preterm birth

© 2024 Jiles M et al. *JAMA Network Open.*

eTable 2: Adjusted[a] odds ratios for occurrence of maternal morbidities by maternal birth country among nulliparous Black-only birthing people in the U.S., National Vital Statistics System Birth Data, 2021. (n =183,600)

Maternal Birthplace	Maternal Morbidity<br>aOR (95% CI)	Maternal Transfusion<br>aOR (95% CI)	Perineal Laceration<br>aOR (95% CI)	Ruptured Uterus<br>aOR (95% CI)	Unplanned Hysterectomy<br>aOR (95% CI)	Admittance to ICU<br>aOR (95% CI)	Low birthweight<br>aOR (95% CI)	Preterm birth<br>aOR (95% CI)
U.S.-born	0.59 (0.54-0.65)	0.82 (0.68-0.99)	0.48 (0.42-0.53)	0.73 (0.24-2.21)	0.39 (0.17-0.89)	0.79 (0.61-1.02)	1.42 (1.35-1.47)	1.30 (1.23-1.36)
Foreign-born	Reference	Reference	Reference	Reference	Reference	Reference	Reference	Reference

[a] Adjusted for: maternal age, maternal education, Hispanic origin, marital status, maternal BMI, month prenatal care began, payment source for delivery, attendant at birth and cigarette use during pregnancy

[b] Due to missing data, n=183,563 for maternal transfusions, n=176,587 for ruptured uterus, n=181,487 for unplanned hysterectomy, and n=183,563 for ICU admittance, n= 183,441 for low birthweight, and n= 183,563 for preterm birth

© 2024 Jiles M et al. *JAMA Network Open.*
