# Supplementary material for: Maternal and Infant Health Outcomes in US-Born and Non–US-Born Black Pregnant People in the US
Source: JAMA Netw Open. 2024 Dec 26;7(12):e2451693. doi: 10.1001/jamanetworkopen.2024.51693 (PMC11672153; doi:10.1001/jamanetworkopen.2024.51693)
Supplement: Supplement 2. — Data Sharing Statement [file jamanetwopen-e2451693-s002.pdf]

Data Sharing Statement

Jiles. Maternal and Infant Health Outcomes in US-born and Foreign-born Black Pregnant People in the US. *JAMA Netw Open*. Published December 23, 2024.
doi:10.1001/jamanetworkopen.2024.51693

Data
Data available: Yes
Data types: Deidentified participant data, Data dictionary
How to access data: https://www.cdc.gov/nchs/data_access/vitalstatsonline.htm
When available: With publication

Supporting Documents
Document types: None

Additional Information
Who can access the data: Publicly-available data
Types of analyses: for any purpose
Mechanisms of data availability: without investigator support
Any additional restrictions: publicly data
